# Special Issue “Molecular Regulation of Drought and Salinity Tolerance in Plants”

**DOI:** 10.3390/ijms262311369

**Published:** 2025-11-25

**Authors:** Guzel Kudoyarova

**Affiliations:** Laboratory of Plant Physiology, Ufa Institute of Biology of the Russian Academy of Sciences, Ufa 450054, Russia; guzel@anrb.ru

Drought and salinity are the main environmental factors reducing plant growth, productivity and crop yields. This is why great efforts are being made to find approaches that increase plant resistance to these stresses. The main reason why drought and salinity have detrimental effects on plants is that they both reduce water availability. Cell expansion, driven by turgor pressure and controlled cell wall changes, is fundamentally dependent on water supply, and its scarcity inevitably inhibits growth, leading to decreased leaf area, photosynthesis and biomass production [1]. Adaptation of plants to water deficit under drought and salinity involves stomatal closure that helps limit water losses and conserve it to support growth. However, decreased stomatal conductance disturbs gas exchange and carbon assimilation [2]. Furthermore, inhibition of photosynthesis is frequently accompanied by excessive production of reactive oxygen species (ROS), which contributes to the detrimental actions of drought and salinity [3]. In addition to impairment of water uptake by high salt concentration, prolonged exposure to this stress factor leads to accumulation of toxic ions (sodium and chlorides) in root and leaf cells, resulting in further inhibition of photosynthesis and uptake of essential elements as well as disturbance of ion homeostasis [4]. Plants cope with the harmful effects of drought and salinity through mechanisms that ensure their resilience. These include, among other mechanisms, adaptive changes in cell wall extensibility [5], accumulation of compatible solutes, which helps to maintain turgor necessary for cell expansion [6], activation of antioxidant system to neutralize ROS and prevent their harmful effects [7], changes in tissue hydraulic conductance depending on deposition of apoplast barriers [8] and activity of water channels aquaporins [9]. Along with the mechanisms responsible for protecting plants from both drought and salinity, salt tolerance is due to adaptive changes in the activity of ion transporters [10]. All of the above mechanisms have been carefully studied, and valuable information has been accumulated. However, many aspects of drought and salinity tolerance remain unclear and require further study. This Special Issue brings together several articles devoted to new aspects of salt tolerance. Although drought tolerance was not specifically addressed in these reports, salt stress serves as a suitable model for studying plant responses to water deficit characteristic of both drought and salinity.

In their review, Lu and Fricke [11] offer a comprehensive analysis of water uptake in the context of the entire plant. The review highlights that few studies distinguish between growing and mature leaf tissues [12,13], although they likely differ in their response to salinity. To protect cytoplasm from toxic ions, plants sequester them into the vacuole, and the extent of salt accumulation in the vacuole depends on the ratio between the vacuolar to extravacuolar space within a cell that differs substantially between different tissues, particularly in leaves. About half of the mesophyll cell volume is occupied by the vacuole, whereas cells within meristems do not have a central vacuole at all. From this point of view, it follows that meristematic cells are potentially the most susceptible to ion toxicity, whereas cells in the mature blade are the least susceptible. However, this pattern is not categorical, and readers are advised to see the details in the review by Lu and Fricke in this SI. Authors of the review point out that, although plants spend more than a third of their lives in darkness, little attention has been paid to how they cope with salt stress during this period. Therefore, the review considers nighttime transpiration and growth. In most reports, daytime transpiration has been assessed while nighttime transpiration has always been assumed to be negligible. However, incomplete stomatal closure and night transpiration have been detected [14,15]. A less negative xylem water potential during the night compared with the day should make it easier for growing leaf cells to take up water. As a result, a higher portion of the water delivered to the shoot may be stored within plants through growth rather than being lost through transpiration [16]. The review considers many other aspects of nighttime mechanisms for salt tolerance, and readers should follow them in the article itself. Furthermore, Lu and Fricke [11] highlight the possible role of symplastic water transport through plasmodesmata in stressed plants. This pathway is the least studied component of transcellular water transport through plant tissues. At the same time, it becomes of great importance under conditions of drought and salinity, when increased deposition of lignin in the Casparian strips reduces water transport through the apoplast [17], and the formation of suberin lamellae in the endodermis negatively affects water transport through the membrane, involving aquaporins [18]. The decline in both apoplast and membrane permeability for water increases the importance of water transport through plasmodesmata, since it is not affected by the formation of suberin lamella.

The article of Wang et al. [19] integrates transcriptomic and metabolomic analyses of indica and japonica rice differing in tolerance to high salinity and alkalinity. Transcriptome analysis identified genes that are particularly upregulated by these stress factors in tolerant rice subspecies (RPY geng). These results were confirmed by metabolome analysis, which allowed identification of key pathways and important factors associated with saline and alkali tolerance. NAC and WRKY transcription factors were significantly and uniquely upregulated in the RPY geng in response to saline–alkaline stress. Furthermore, these transcription factors were highly co-expressed with the *GPX* gene encoding glutathione peroxidases that act as cellular antioxidants by protecting against oxidative damage from ROS. The results are in accordance with the data showing involvement of the *GPX* gene in salt tolerance [20]. In the article of Wang et al. [19], it was found that the MDA concentration (indicator of oxidative damage) was markedly higher in Chao2R (more susceptible genotype) than in RPY geng, which confirmed better protection of RPY geng from ROS. Plant hormone signal transduction pathways were upregulated in RPY geng, confirming the importance of hormones in the control of saline–alkaline stress. Out of them, *BRI1*, which encodes a key component of the brassinosteroid signaling pathway, was identified as a hub gene, i.e., a gene with a large number of interactions in a gene network [21]. *Aux/IAA* gene encoding a repressor of auxin-responsive gene expression [22] was rapidly and specifically induced by saline–alkaline stress in RPY geng. A similar response was detected in the case of *GID1* encoding receptor that binds gibberellins [23] and *JAZ* gene encoding co-receptors and transcriptional repressors of jasmonic acid [24].

The paper by Shahzad et al. [25] explores the importance of the Na^+^ exclusion mechanism mediated by SOS1 (plasma membrane-localized Na^+^/H^+^ antiporter) for the development of salt tolerance in rice plants. The *SOS1* gene encoding this antiporter is responsible for active Na^+^ exclusion from cells [26,27]. To achieve their goal, Shahzad et al. [25] compared the expression of the *SOS1* gene in two contrasting pairs of cultivated and wild rice species and related the results to Na^+^ and H^+^ fluxes measured using a non-invasive ion-selective vibrating microelectrode. Pharmacological experiments, demonstrating inhibition of Na^+^ efflux in the root elongation zone (EZ) by amiloride (an inhibitor of the plasmalemma Na^+^/H^+^ exchangers [28,29]), confirmed involvement of these exchangers in Na^+^ efflux. In both cultivated and wild rice groups, amiloride-sensitive Na^+^ efflux was higher in tolerant genotypes. Lui et al. [30] reported that the increase in *OsSOS1* expression induced by salinity was higher in the salt-tolerant cultivar Reiziq compared to other rice cultivars. However, in the study by Shahzad et al. [25], no significant difference in *SOS1* expression was found under salinity stress in cultivated rice (IR1-tolerant and IR29-sensitive). Authors suggest that in their experiments operation of SOS1 in cultivated rice is regulated at a post-translational rather than transcriptional level. The Na^+^/H^+^ exchanger activity was 2–3 times higher in cultivated rice than in wild rice, suggesting that cultivated rice relies more on the Na^+^ exclusion mechanism to cope with salt stress than wild rice. Wild rice species likely use other mechanisms that conserve energy [31]. In conclusion, it is assumed that Na^+^ extrusion is more essential for cultivated rice, which is due to domestication strategy and selection.

The paper by Zhao et al. [32] reports on the identification of the NAC transcription factors and their function in response to salinity in lotus (*Nelumbo nucifera*). Plants of this species are perennial aquatic herbs that are important model plants in horticulture and are widely distributed and used in China. Lotus genome has been sequenced, and genes encoding several transcription factors (TF) have been identified and studied [33,34,35]. However, the gene family of NAC TF has not yet been comprehensively investigated in lotus. This family of TF plays an important role in plant salinity tolerance by regulating various stress-responsive genes that help plants cope with salt stress [36,37]. qRT-PCR analysis demonstrated that *NAC016*, *NAC025*, and *NAC070* genes were up-regulated by NaCl treatment of lotus. Cis-element analysis demonstrated that *NAC* genes are responsive to plant hormones, including abscisic acid (ABA). ABA is an important phytohormone involved in the regulation of plant growth, development, and stress responses [38]. The presence of cis-elements responsible for responsiveness to ABA suggests that *NAC* genes may be regulated by the ABA-related transcription factors. qRT-PCR analysis described in the paper by Zhao et al. [32] confirmed that lotus *NAC* genes were induced under ABA treatment, while the co-expression network indicated that functions of NAC genes and ABA signaling are closely linked. These results are consistent with the data showing that OsNAC45 was involved in ABA response and salt tolerance in rice [39]. In total, more than 90 *NAC* genes have been identified in lotus, which may be involved in responses to biotic and abiotic stresses and would be the key candidate genes for further functional research by modern genetic and biological techniques.

Paper by Efimova et al. [40] describes how melatonin treatment protects stolon formation in salt-stressed potato plants and that its effects depend on photochemical function of Photosystem II, ionic homeostasis, and activity of the antioxidant system. Melatonin is able to protect agricultural plants from the harmful effects of various stressors, including salinity [41,42,43]. The aim of the study by Efimova et al. was to test whether short-term treatment of potato plants with melatonin could have a protective effect on growth processes and stolon formation during upcoming salt stress. It was shown that short-term treatment of potato plants with melatonin reduced the negative effects of NaCl on stolon formation and ionic status, but did not affect expression of Na^+^/H^+^ antiporter genes localized in the vacuolar membrane (NHX1–NHX3), while the increase in the level of the plasma membrane antiporter (SOS1) was not statistically significant. However, it has been suggested that melatonin likely stimulates the activity of SOS1, which is known to export sodium ions from the cytoplasm to the apoplast [44]. This may be due to the indirect effect of melatonin on ion homeostasis. Thus, melatonin was shown to stimulate energy metabolism under salt stress conditions, thereby contributing to the maintenance of plasma membrane H^+^-ATPase activity and K^+^/Na^+^ homeostasis in sweet potato [45]. Melatonin effectively reduced the accumulation of lipid peroxidation products. This was unlikely due to its influence on antioxidant enzymes, since neither SOD nor peroxidase was activated by melatonin. It exerted positive effects on low-molecular-weight antioxidants: proline, flavonoids, and carotenoids, which contributed to decreasing oxidative stress.

Although long-term reduction in stomatal conductance in salt-stressed plants impairs gas exchange and carbon assimilation [2], short-term stomatal closure allows time for plants to adapt to salinity-induced water deficits. Therefore, the mechanisms that ensure rapid stomatal closure at the onset of salt treatment are of great interest, but remain poorly understood. The aim of the work of Sharipova et al. [46] was to test for any involvement of aquaporins (AQPs) in stomatal closure in salt-stressed barley plants. The changes in the level of AQPs in the cells were detected with the help of an immunohistochemical technique using antibodies against HvPIP2;2. In parallel, leaf sections were immunostained for abscisic acid (ABA). The effects of salinity on leaf HvPIP2;2 levels, stomatal and leaf hydraulic conductance, were compared to those of exogenously applied ABA. Salinity reduced the abundance of HvPIP2;2 in the cells of the mestome sheath, resulting in a decline in the hydraulic conductivity, transpiration, and ABA accumulation. The effects of exogenous ABA differed from those of salinity. This hormone decreased transpiration but increased the shoot hydraulic conductivity and abundance of HvPIP2;2. The difference in the action of the exogenous hormone and salinity may be related to the difference in the ABA distribution between leaf cells: this hormone accumulated mainly in the mesophyll of salt-stressed plants and in the cells of the bundle sheaths of ABA-treated plants. Accumulation of ABA in bundle sheaths of plants treated with these hormones explains increased abundance of HvPIP2;2 and hydraulic conductance, since this hormone is known to influence aquaporins in this way [47]. The obtained results suggest the following succession of events: salinity decreases water flow into the shoots due to the decreased abundance of PIP2;2 and hydraulic conductance, while the decline in leaf hydration leads to the production of ABA in the leaves and stomatal closure.

The present review of the papers published in this Special Issue shows that they describe new and interesting aspects of drought and salt tolerance revealed by studying salt stress as a model for revealing plant adaptation to water deficit.
